# Viekira Pak Induced Fatal Lactic Acidosis: A Case Report of an Unusual Side Effect

**DOI:** 10.1155/2016/8627139

**Published:** 2016-12-01

**Authors:** Molham Abdulsamad, Ariyo Ihimoyan

**Affiliations:** Department of Medicine, Division of Gastroenterology, Bronx Lebanon Hospital Center, 1650 Selwyn Avenue, Suite No. 10C, Bronx, NY 10457, USA

## Abstract

Viekira Pak is a new direct-acting antiviral agent that has an excellent efficacy in treating patients with chronic HCV. FDA released a safety warning that Viekira Pak can cause serious liver injury mostly in patients with underlying advanced liver disease. We report the first case of fatal lactic acidosis presenting 3 days after initiating therapy with Viekira Pak. Although it is very hard to precisely determine the cause of lactic acidosis, our case highlights an unusual side effect that ensued after starting the medication. Given the complexity of drug-drug interactions that can happen with the new direct-acting antiviral agents and the paucity of data regarding coadministration and methods of monitoring, a thorough review should be pursued prior to initiating these medications.

## 1. Introduction

It is been estimated that more than 5 million people in the United States are chronically infected with hepatitis C virus (HCV) [1]. The two major long-term complications of this infection are liver cirrhosis and hepatocellular carcinoma (HCC). So far, this is the only chronic viral infection of the liver that can be completely cured with antiviral therapy [2] especially after the invention of the new direct-acting antiviral agents (DAAs). These agents aim to target HCV NS3/4 protease, NS5B polymerase, and NS5A proteins [3]. Current AASLD guidelines recommend treating all patients with chronic HCV infection except those with short life expectancies as it proved to reduce mortality and complications of liver disease [4]. In December 2014, Viekira Pak (*Ombitasvir*, an NS5A inhibitor,* Paritaprevir*, NS3/4A protease inhibitor,* Dasabuvir*, NS5B polymerase inhibitor, and* Ritonavir*, a CYP3A inhibitor to increase plasma drug concentrations of Paritaprevir) [5] was approved for the treatment of HCV. It combines three DAAs with distinct mechanisms of action to target HCV at multiple steps in the viral lifecycle [6]. Up to this moment, thousands of Viekira Pak prescriptions have been dispensed. The most reported adverse reactions were nausea, pruritus, and insomnia [7]. In October 2015, the FDA released a safety warning that Viekira Pak can cause serious liver injury mostly in patients with underlying advanced liver disease [8]. Viekira Pak is safe to be used in patients with chronic kidney disease and no dose adjustment needed for patients with low GFR. Upon review of the literature, only one case report of Viekira Pak induced lactic acidosis and shock has been reported [9] where the patient improved after hemodialysis. We report the first case of fatal lactic acidosis and shock and rapid multiorgan failure 3 days after initiating Viekira Pak.

## 2. Case Report

A 69-year-old man known to have chronic hepatitis C viral infection from prior intravenous illicit drug use, genotype 1b with viral RNA of 23,048,964 IU/ML, and treatment naive has other medical comorbidities of hypertension, chronic obstructive pulmonary disease, benign prostatic hyperplasia, methadone dependence, and chronic kidney disease stage IV. His comorbid conditions were chronically stable with well controlled blood pressure and stable kidney function over the last few years. He was evaluated in the gastroenterology clinic for HCV and deemed a candidate for the new antiviral therapy. He had a liver biopsy three years ago showing bridging fibrosis. His most recent fibrosis score revealed F4 Metavir just weeks prior to initiating therapy which indicates possible underlying liver cirrhosis with a Child-Pugh score class A (total score = 5, based on serum bilirubin = 0.6 mg/dL, albumin = 4.1 g/dL, INR = 1.2, and no ascites or encephalopathy). Ultrasound of the abdomen showed his liver measures 17 cm in length and demonstrates normal contour and echogenicity. Spleen size was normal with no definite radiological evidence of liver cirrhosis. His home medications included tamsulosin, albuterol and ipratropium inhalers, sodium polystyrene, sodium bicarbonate, nifedipine, metoprolol, hydralazine, furosemide, and low dose Aspirin. After careful review of potential drug-drug interactions, no major contraindication or serious drug-drug interaction precluding initiation of the medication was predicted based on a detailed review of the drug's full prescribing information; see Table 1 for a summary of the drug-drug interactions. The doses of nifedipine and furosemide were not changed but planned for close monitoring of his BP and further adjustment based on BP changes. He was started on Viekira Pak and planned for 12 weeks of therapy. Three days after initiating therapy he presented to the emergency department with nausea, vomiting, and shortness of breath for one-day duration. He denied any abdominal pain, diarrhea, fever, or jaundice, no chest pain or palpitations. Review of systems was negative. He denied any alcohol or illicit drug use. On physical exam, his blood pressure was 116/66 mmHg, heart rate of 59 bpm, respiratory rate 20, temp 98°F, and O_2_ saturation 86% on room air. He was in mild respiratory distress, had no scleral icterus, and was alert and oriented x3, and otherwise his physical examination was unremarkable. Laboratory studies revealed a venous PH of 7.22, potassium of 7.3 mEq/L, WBC of 10.9 × 10^6^/L, and no left shift. Measured anion gap = 29 mEq/L, BUN/Cr = 71/5.9 mg/dL, normal LFTs except for AST are 56 U/L, and an international normalization ratio (INR) is 1.3. Lactic acid is 14.7 mmoles/L, negative troponins. Portable CXR showed possible atelectasis within the bilateral lower lungs and small to moderate size right pleural effusion unchanged from prior X-rays. His EKG showed sinus rhythm with T wave inversion across multiple leads. He was given multiple cycles of intravenous calcium, insulin with dextrose and bicarbonate, and repeated potassium of 4.6 mEq/L. He was placed on a noninvasive positive pressure ventilation (NIPPV), central dialysis catheter placed and started on hemodialysis. Later he was intubated and started on vasopressors. Despite aggressive resuscitation measures, he had cardiac arrest and died on a Friday.

## 3. Discussion

In this case, our patient is known to have advanced stage chronic kidney disease which seems to be stable for many years and is being managed with medical therapy to control hyperkalemia with no indication to start him on long-term hemodialysis. He presented with acute kidney injury causing hyperkalemia which is likely part of an acute event causing multiorgan failure rather than just worsening of his chronic kidney disease that would not explain his severe lactic acidosis. Also, he did not respond to hemodialysis although it was short duration before he had a cardiac arrest. There were no clinical signs or symptoms of underlying infection and his WBC count was just borderline. His cardiac markers were normal and EKG did not show evidence of ongoing acute coronary syndrome or arrhythmia. There were no clear signs of organ hypoperfusion based on his normal blood pressure, intact mental status, and no clinical evidence of ongoing shock. Our patient did not have any hepatotoxicity, based on his normal ALT, to contribute to his lactic acidosis despite his advanced liver disease.

All four drugs of Viekira Pak are highly protein bound (>97%) with an average half-life of 4-5 hours. Ombitasvir is metabolized by amide hydrolysis followed by oxidative metabolism. Paritaprevir and ritonavir are predominately metabolized by CYP3A4. Dasabuvir is metabolized by CYP2C8 and CYP3A4 to a lesser extent. CYP3A4 is an enzyme mainly found in the liver and oxidizes drugs so that they can be eliminated from the blood [10]. None of our patient's medications had CYP3A4 inhibitor activity except ritonavir which is part of Viekira Pak.

Moreover, none of these drugs is metabolized or excreted by the kidney and none is dialyzed by hemodialysis. In our case, hemodialysis was initiated to exclude the possibility of his clinical deterioration due to worsening kidney disease. Noteworthy, hemodialysis may theoretically help if drug-drug interaction is suspected and the toxic agent is eliminated by hemodialysis.

Initiation of ritonavir in a patient who is taking medications metabolized by CYP3A may increase the plasma concentrations of these medications. These interactions may lead to clinically significant adverse reactions, potentially leading to severe or fatal events; hence close monitoring of these side effects is warranted. Nifedipine and tamsulosin are substrates of CYP3A4 and inhibition by ritonavir can cause potential hypotension and cardiac arrhythmias. Coadministration is not absolutely contraindicated but dose reduction and/or clinical monitoring is recommended when coadministered. Furthermore, Flomax® which is the brand name for tamsulosin should not be used in combination with a strong CYP3A inhibitor.

Similarly, coadministration of furosemide and Viekira Pak showed a potential increase in furosemide activity; hence caution is warranted and clinical monitoring is recommended.

Mitochondria play a crucial role in human bodies to supply energy to cells in the form of ATP [11]. Many drugs like antiretrovirals, metformin, NSAIDs, local anesthetics, neuroleptics, chemotherapeutics, and others are reported to cause mitochondrial dysfunction leading to cell death and necrosis [12]. Such toxicity usually manifests with elevated serum lactic acid and patients will usually have symptoms of fatigue, nausea, and vomiting [11]. The disease course can often be severe and fatal if not early detected and the triggering agent was discontinued.

Lactic acidosis is one of the major causes of metabolic acidosis. Type A is due to tissue hypoperfusion as seen in septic shock, cardiac failure, and hypovolemia. Type B is seen in toxin-induced impairment of cellular metabolism [13]. Based on our patient's clinical presentation, the lack of any evidence of common causes of lactic acidosis like sepsis or heart failure, and his normal blood pressure upon presentation, we contemplate the possibility of mitochondrial dysfunction causing severe, persistent lactic acidosis that ensued shortly after starting Viekira Pak, though this it is hard to be proved in our case given the rapid course.

Our patient was on three different medications that have antihypertensive effects (furosemide, nifedipine, and tamsulosin) which have a potential drug-drug interaction with Viekira Pak causing increased antihypertensive effect but our patient did not have hypotension upon presentation.

DAAs including Viekira Pak are safe and potent drugs with excellent antiviral effect [14]. There are many reported drug-drug interactions; hence it is imperative to check the manufacturer's label prior to initiating the treatment. Despite our careful review of the patient's medications and potential drug-drug interactions with Viekira Pak, we did not anticipate any serious interactions like our patient's presentation that warrant discontinuing any of his medications but to closely monitor the patient and consider dose reduction of some of his medications if any signs of toxicity emerge. Treatment of hepatitis C is highly encouraged but we believe that our case should highlight the importance of possible side effects that may not be expected in a newly emerging potent drug especially in the case of potential drug-drug interactions that needs a better precise monitoring protocol.

Lactic acidosis had not been reported as a potential side effect of Viekira Pak and one cannot predict which patient is at risk of this complication. Our patient had both advanced kidney and liver disease and was on multiple antihypertensive medications that have a potential interaction with Viekira Pak; hence careful monitoring for such side effect is particularly suggested in such patients. Further future studies are needed on how to accurately monitor these patients and how to monitor those potential drug-drug interactions.

## Figures and Tables

**Table 1:** Summary of the anticipated drug-drug interactions between the patient's current medications and Viekira Pak.

Drug	Drug interactions with Viekira Pak
Low dose Aspirin	No interaction expected
Albuterol inhaler	No interaction expected
Ipratropium bromide inhaler	No interaction expected
Sodium bicarbonate	No interaction expected
Sodium polystyrene	No interaction expected
Furosemide	Potential interaction
Hydralazine	No interaction expected
Metoprolol	No interaction expected
Nifedipine	Potential interaction
Tamsulosin	Potential interaction
